# Effect of the Hydrophilic-Hydrophobic Balance of Antigen-Loaded Peptide Nanofibers on Their Cellular Uptake, Cellular Toxicity, and Immune Stimulatory Properties

**DOI:** 10.3390/ijms20153781

**Published:** 2019-08-02

**Authors:** Tomonori Waku, Saki Nishigaki, Yuichi Kitagawa, Sayaka Koeda, Kazufumi Kawabata, Shigeru Kunugi, Akio Kobori, Naoki Tanaka

**Affiliations:** Faculty of Molecular Chemistry and Engineering, Kyoto Institute of Technology, Gosyokaido-cho, Matsugasaki, Sakyo-ku, Kyoto 606-8585, Japan

**Keywords:** peptide, nanofibers, poly(ethylene glycol), antigen delivery, immune stimulation

## Abstract

Recently, nanofibers (NFs) formed from antigenic peptides conjugated to β-sheet-forming peptides have attracted much attention as a new generation of vaccines. However, studies describing how the hydrophilic-hydrophobic balance of NF components affects cellular interactions of NFs are limited. In this report, three different NFs were prepared by self-assembly of β-sheet-forming peptides conjugated with model antigenic peptides (SIINFEKL) from ovalbumin and hydrophilic oligo-ethylene glycol (EG) of differing chain lengths (6-, 12- and 24-mer) to investigate the effect of EG length of antigen-loaded NFs on their cellular uptake, cytotoxicity, and dendritic cell (DC)-stimulation ability. We used an immortal DC line, termed JAWS II, derived from bone marrow-derived DCs of a C57BL/6 p53-knockout mouse. The uptake of NFs, consisting of the EG 12-mer by DCs, was the most effective and activated DC without exhibiting significant cytotoxicity. Increasing the EG chain length significantly reduced cellular entry and DC activation by NFs. Conversely, shortening the EG chain enhanced DC activation but increased toxicity and impaired water-dispersibility, resulting in low cellular uptake. These results show that the interaction of antigen-loaded NFs with cells can be tuned by the EG length, which provides useful design guidelines for the development of effective NF-based vaccines.

## 1. Introduction

Peptide-based synthetic vaccines have attracted a significant amount of attention as a new generation of vaccines, because of their safety benefits and ease of production when compared with that of conventional whole pathogen-based vaccines [1,2]. However, poor immune responses are induced when only minimal antigenic epitopes are used without combining suitable adjuvants (immune stimulants), which are sometimes toxic. Nanocarrier-based delivery systems are a promising approach to overcome those drawbacks of peptide vaccines. In designing the nanocarrier, it is important to consider the interaction between the nanocarrier surface and cells, such as antigen presenting cells (APCs).

Over the past few decades, various nanocarriers have been developed, including liposomes [3,4,5], polymeric nanoparticles [6,7,8], and polymeric micelles [9]. In many of these systems, building block molecules for the construction of nanocarriers are first synthesized and then combined with antigenic peptides via several procedures, including nanomaterial formation and loading of antigenic peptides (encapsulation, chemical immobilization or physical adsorption), to give a nano-formulation. Recently, the use of antigenic peptides that are pre-conjugated to self-assembly motifs has attracted attention as an easier and simpler procedure to produce nano-formulations [10,11]. This self-assembly approach ensures highly efficient drug loading without laborious procedures or the use of synthetic components, which sometimes exhibit toxicity. In addition, because the resulting nanostructures consist of a single component, the physicochemical and structural features of these nanostructures can be simply tuned by the design of the building block peptide, and variation in drug loading efficiency among different nanostructures can be eliminated.

Among the various molecular blocks (e.g., lipids [12,13,14] and hydrophobic polymers [15,16]) used to assemble antigenic peptides into nanostructures, β-sheet-forming-peptides are extremely attractive because: (i) They can assemble in aqueous solution to give nanofibers (NFs) with highly regulated structures, even when functional molecules with a relatively large molecular weight are conjugated; (ii) the resulting well-ordered β-sheet structures allow the integration of antigens at high density; and (iii) they are relatively easy to synthesize and have high biocompatibility. These advantages make NF-vaccines a good alternative to traditional vaccines. Immune induction by NFs formed from antigenic peptides conjugated to β-sheet-forming-peptides have been reported [17,18,19,20,21]. For example, Rudra et al. reported that NFs composed of an antigenic epitope peptide conjugated to self-assembling peptide Q11 were subcutaneously administered, and elicited a strong antibody response [17]. They have also demonstrated that the β-sheet peptide NF system can be applied to various types of antigens, including a malaria epitope [18], a *Staphylococcus aureus* epitope [19] and a tumor-associated antigen MUC1 glycopeptide [20]. However, fundamental studies on how the hydrophilic-hydrophobic balance of NF components affects their cellular interaction—including cellular uptake, cytotoxicity, and immune stimulation response—has not been reported. Recently, studies on other particulate systems reported that surface hydrophobicity is an important factor for determining cellular response [22,23,24,25,26,27,28,29,30,31,32]. In addition to cellular internalization and nontoxicity (i.e., safety), the ability of nanocarriers to stimulate an immune response is an essential property in nanocarrier-based vaccine applications, because uptake of nanocarriers containing an antigen by APCs that do not induce an immune response may lead to unwanted tolerance toward the antigen. Thus, to design nanomaterial-based vaccines that elicit strong immunity without toxicity using a β-sheet assembly system requires a clear understanding of how the hydrophilic-hydrophobic balance of NF components affects their cellular interactions and response.

**Figure 1:**
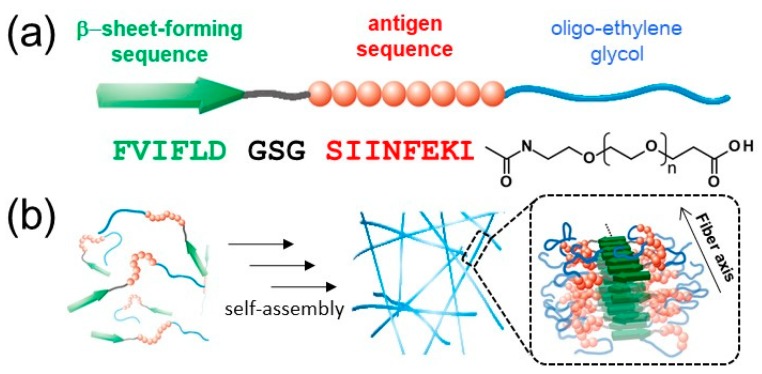
(**a**) Design of the building block peptides (EG*_n_*) that are composed of a β-sheet-forming sequence (FVIFLD), a flexible-linker block (GSG), a model antigen sequence (SIINFEKL from OVA), and oligo-ethylene glycol. (**b**) Schematic illustration of the self-assembly process for nanofiber formation and the proposed model of highly antigen-loaded nanofibers based on previous structural study [36]. The schematic illustration was created by modification of Figure 1 and Figure 2 of reference [35].

In previous work, we reported the preparation of antigen-loaded NFs by exploiting the self-assembly of β-sheet peptides [33,34] conjugated to antigenic peptides and hydrophilic chains, such as oligo-ethylene glycol (EG) [35,36,37]. MHC class I restricted epitope (SIINFEKL) from ovalbumin was selected as a model antigenic peptide. In addition, the structure of the NFs was analyzed in detail by various techniques, including wide-angle X-ray diffraction (WAXD), small-angle X-ray scattering (SAXS), Fourier transform infrared spectroscopy (FT-IR), circular dichroism (CD), transmission electron microscopy (TEM), and atomic force microscopy (AFM). Interestingly, structural analysis revealed that the shape of the NFs is rectangular, rather than a cylinder-like structure observed for filament micelles, possibly because of the lamination structures of β-sheets. Based on this finding, the structural model was proposed as shown in Figure 1b, which shows that the surface of the NFs is not covered with EG chains homogeneously [35,36]. Thus, we hypothesized that the EG chain length is an important parameter for tuning the cellular interactions of NFs, including cellular uptake, cytotoxicity, and immune stimulation response.

In this study, the effect of the EG chain length in building block molecules, which form peptide NFs, on their cellular interaction was investigated. The self-assembling behavior of three kinds of building block peptides with different EG lengths was evaluated by determining their critical aggregation concentration (CAC) and the critical concentration for nanofiber formation (CFC). The structures of the resulting NFs were analyzed by TEM and CD, and their surface hydrophobicity was evaluated using a hydrophobic fluorescence probe. Cellular uptake, cytotoxicity, and immune stimulation ability of the three kinds of NFs were examined in vitro using DCs. In addition, interaction of cells with micelle-like aggregates that were composed of the same building blocks as the NFs were also investigated. Cellular interaction of the NFs was found to be significantly dependent on EG length, whereas that of micelles was independent of EG length. Notably, uptake by DC of NFs composed of EG with a moderate length was effective, and the NFs activated DC without exhibiting significant cytotoxicity. The findings provide useful design guidelines for the development of effective nanofiber-based vaccines.

## 2. Results

### 2.1. Self-Assembly Behavior of EG_n_ Peptides

We have reported previously the preparation of NFs in aqueous solution by heat-treatment of the EG_12_ peptide (EG*_n_*, where *n* is the length of the EG chain), which is composed of a β-sheet-forming sequence, an antigen sequence, and a hydrophilic oligo-ethylene glycol (12-mer). In this study, three building block peptide amphiphiles with different EG lengths (6-mer, 12-mer, and 24-mer, which are termed EG_6_, EG_12_, and EG_24_, respectively) were prepared for assessing the effect of EG length on cellular uptake of NFs, cytotoxicity, and immune stimulatory activities.

**Figure 2:**
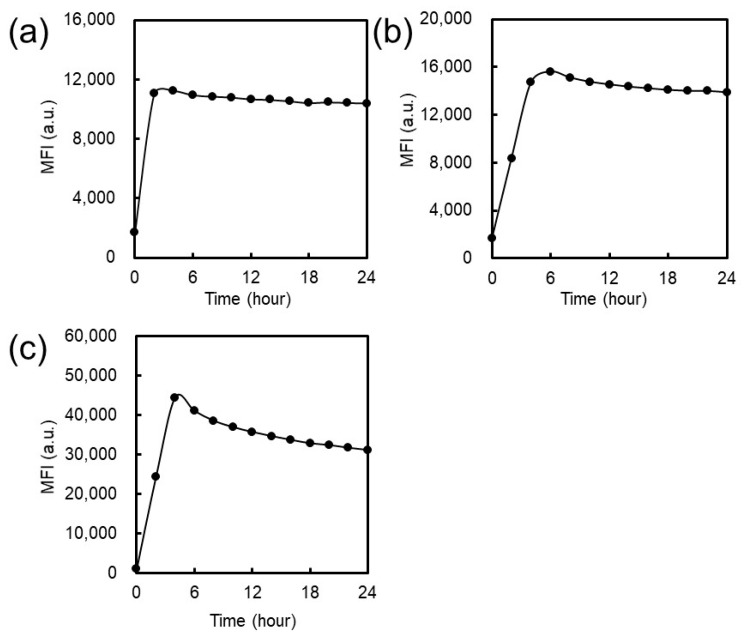
Time-dependent change in the ThT fluorescence intensity of solutions containing the (**a**) EG_6_ peptide, (**b**) EG_12_ peptide, and (**c**) EG_24_ peptide when incubated at 37 °C.

Initially, we investigated the effect of EG length on the self-assembly behavior of these peptides by estimating the CFC of each peptide. EG*_n_* peptides were incubated in the presence of the thioflavin T (ThT) dye at 300 μM in phosphate buffered saline (PBS) containing 5% dimethylsulfoxide (DMSO) at 37 °C for 24 h, and a time course of change in ThT fluorescence was measured. The ThT assay is often used to monitor the growth of amyloid-like nanofibers from their component peptides or proteins. A remarkable increase in ThT fluorescence was observed, indicating the formation of NFs by each peptide (Figure 2 and Figure S1). After a certain period of time, the intensity of the ThT fluorescence reached a plateau value. The systems were in a dynamic equilibrium between fibrils and the peptide monomer when the ThT fluorescence intensity reached a plateau value. The concentration of the free peptide monomer at equilibrium corresponds to the CFC [38,39]. The peptide concentration in the supernatant following ultracentrifugation of the EG*_n_* peptide solutions incubated at 300 μM and 37 °C for 24 h provided estimates of the CFC values, which were 96.0 ± 3.6 μM for EG_6_, 72.4 ± 3.7 μM for EG_12_, and 83.8 ± 1.0 μM for EG_24_.

Below the CFC, EG*_n_* peptides could either form spherical micelle-like structures or exist as isolated molecules in water because of their amphiphilic structures [40]. The CAC was determined for EG*_n_* peptides in PBS (pH 7.4) using the pyrene 1:3 method to gain information on the association state of EG*_n_* peptides at relatively low concentrations. The CAC was estimated to be 16.6 μM for EG_6_, 21.7 μM for EG_12_, and 29.9 μM for EG_24_ (Figure S2). The CAC value increased as the EG length increased. Because CAC values were smaller than the CFC values, EG*_n_* peptides self-assembled into spherical micelle-like structures over the concentration range between the CAC and the CFC, and existed as monomers at concentrations below the CAC.

### 2.2. Structural Characterization of EG_n_ Nanofibers

NFs consisting of EG*_n_* peptides were prepared by incubation of the peptide solution in PBS containing 5% DMSO at 60 °C for 24 h. The incubation was carried out at a higher temperature in this experiment when compared with that of the ThT assay to accelerate NF formation. The resulting NFs were characterized by TEM, CD, ς-potential measurements, and the 8-anilino-1-naphtalene sulfonic acid (ANS) assay.

**Figure 3:**
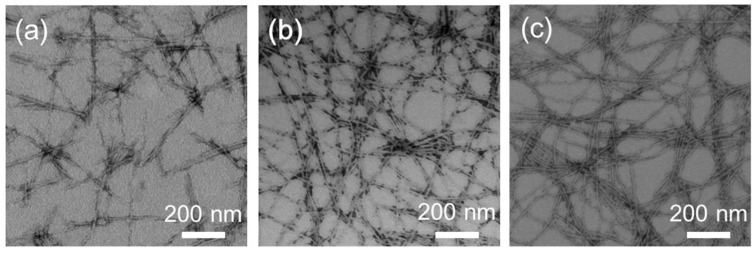
Negatively-stained TEM images of the nanofibers (NFs) obtained by incubation of the (**a**) EG_6_ peptide, (**b**) EG_12_ peptide, or (**c**) EG_24_ peptide at a concentration of 300 μM for 24 h in PBS at 60 °C.

**Figure 4:**
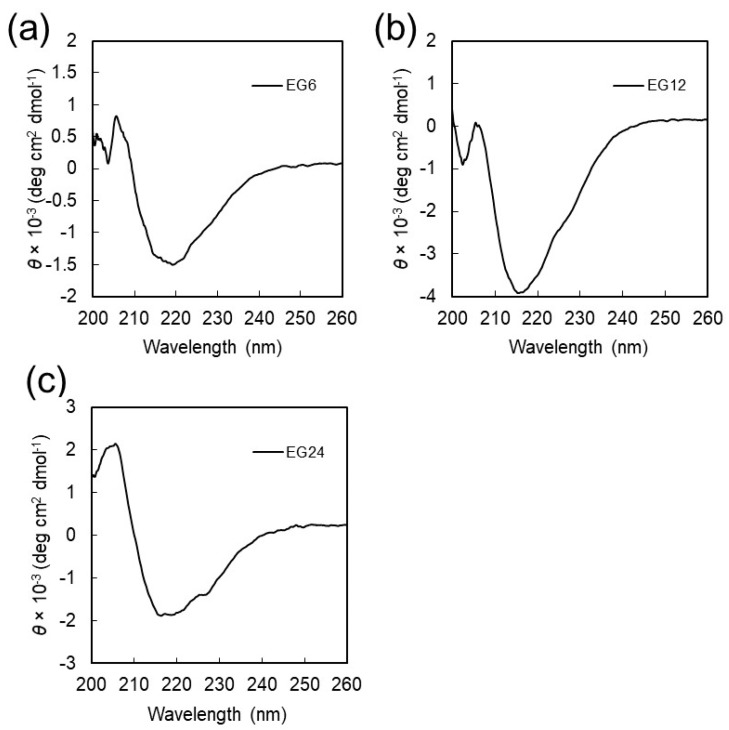
CD spectra of EG*_n_* NFs measured in PBS at room temperature. The EG*_n_* NFs were prepared by incubation of EG*_n_* peptide solutions in PBS at 60 °C for 24 h. (**a**) EG_6_ NFs, (**b**) EG_12_ NFs, and (**c**) EG_24_ NFs.

TEM images revealed that all peptides successfully formed NFs with a homogenous distinct width of ca. 6–8 nm and lengths of several micrometers (Figure 3). The values of the ς-potentials were −33.2 ± 10.7 mV for EG_6_ NFs, −33.2 ± 10.7 mV for EG_12_ NFs, and −32.4 ± 9.6 mV for EG_24_ NFs, showing that the surface of the NFs were negatively charged. The ANS assay was performed to obtain information on the surface hydrophobicity of the NFs [41,42]. The peaks shifted toward shorter wavelengths, and the intensities of the signals increased significantly for all NFs (Figure S3). These observations indicate that there are hydrophobic domains on the surface of the peptide NFs. CD was used to examine the secondary structures adopted by peptides in the NFs. The characteristic negative Cotton peak at 217 nm was observed for the three EG*_n_* NFs, showing that the peptides adopt a β-sheet conformation (Figure 4) [43]. These results indicate that the three NFs possess a similar secondary structure regardless of EG length.

### 2.3. Cellular Uptake, Cytotoxicity, and Maturation of DCs

We investigated the effect of the EG length of nanofibers on their cell association, cytotoxicity, and DC stimulatory activities. The peptide NFs obtained by incubation at a concentration of 1.5 mM and 60 °C for 24 h were used. The length of the NFs was controlled to be 230–260 nm by an extrusion procedure using a membrane filter with a diameter of 450 nm (Figures S4 and S5). Information about the dispersion state of NFs in water was determined by dynamic light scattering (DLS) measurements of EG_6_ NFs, EG_12_ NFs, and EG_24_ NFs. The DLS histograms for EG_12_ and EG_24_ NFs exhibited a unimodal peak, with average diameters of 203.7 ± 119.7 nm for EG_12_ NFs, and 116.6 ± 68.2 nm for EG_24_ NFs. These values are inconsistent with the size estimates from TEM images and this is possibly because of their non-spherical morphology. Nonetheless, these unimodal histograms clearly indicate that EG_12_ and EG_24_ NFs exist as isolated NFs without aggregation in aqueous media (Figure S6). In contrast, the DLS histogram for EG_6_ NFs exhibited two peaks with sizes of 135.4 ± 14.5 nm and 3498.3 ± 389.0 nm, indicating that EG_6_ NFs formed large aggregates. The secondary aggregation of the NFs may be caused by association of surface-exposed hydrophobic domains on the NFs. Longer EG chains effectively prevent these interactions, yielding highly dispersed, stable EG_12_ and EG_24_ NFs. Conversely, the 6-mer EG is not sufficiently long to prevent secondary aggregation of the peptide NFs, resulting in the observed large aggregates. To compare the behaviors of NFs, the cellular uptake, cytotoxicity, and DC stimulatory activities of non-heat-treated EG*_n_* peptides (non-fiber) were also evaluated. The samples for these experiments were prepared by direct dissolution of the peptides in medium at a given concentration to avoid self-assembly into NFs. 

#### 2.3.1. Cellular Association

We evaluated the effect of EG length on the cellular association of EG*_n_* NFs. The fluorescence-labeled EG*_n_* NFs were incubated with JAWS II cells for 1 h at 37 °C. JAWS II cells are an immortalized immature DC line that was established from bone marrow cultures of C57BL/6 p53-knockout mice [44,45]. The amount of NFs associated with the cells was evaluated by flow cytometry (FCM). For comparison, cellular association of non-heat-treated EG*_n_* peptides was also performed.

**Figure 5:**
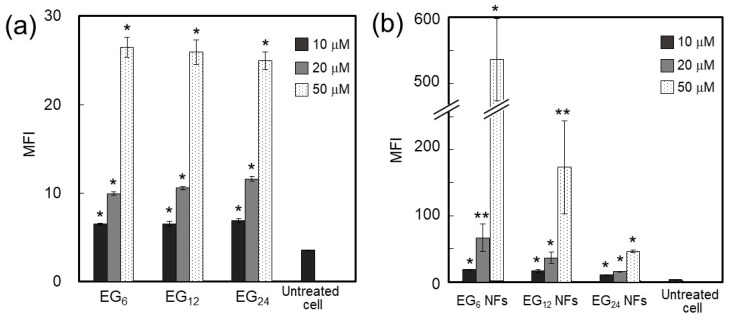
Evaluation of the cellular association of various (**a**) EG*_n_* peptides and (**b**) EG*_n_* NFs labeled with fluorescein using FCM. Mean fluorescence intensity of the treated JAWS II cells is shown. Cellular treatment was performed by incubating cells with peptides or NFs in serum-free medium at 37 °C for 2 h. Each point is the mean ± SD (*n* = 3). * *p* < 0.01, ** *p* < 0.01 compared to untreated cell.

As shown in Figure 5a, the intensity of fluorescence signals from cells incubated with EG*_n_* peptides (non-fiber) increased as the concentration of the peptides increased. This trend was common to all peptides examined. Comparison of the fluorescence intensity of the three EG*_n_* peptides at the same concentration revealed that they were very similar, indicating that EG length had no effect on cellular association of the peptides. In contrast, cellular association of EG*_n_* NFs was influenced noticeably by EG length (Figure 5b). The amount of associated EG*_n_* NFs was larger as the EG length decreased. This trend was more apparent as the concentration of the peptide increased. This result indicates that longer EG chains may prevent interactions between cells and NFs.

**Figure 6:**
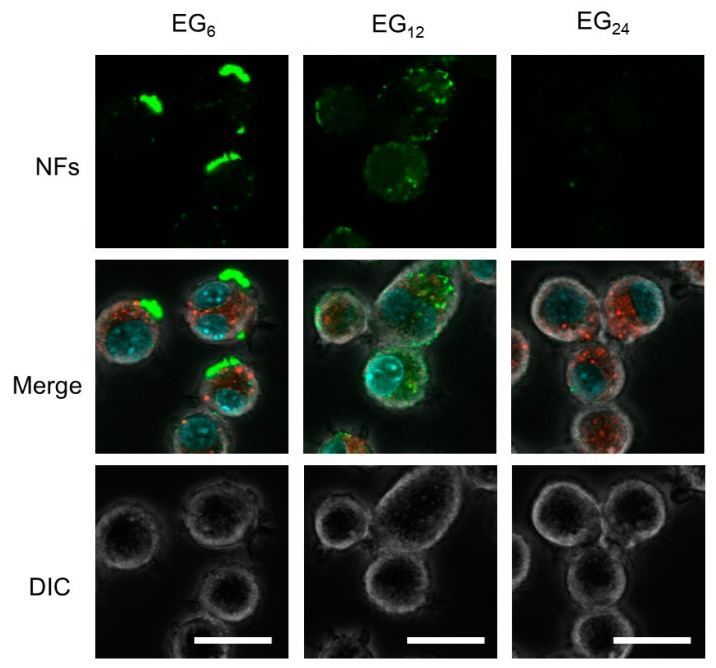
CLSM images of JAWS II cells treated with various EG*_n_* NFs labeled with fluorescein. JAWS II cells were incubated with EG*_n_* NFs at 37 °C for 2 h in serum-free medium. After incubation, the cells were treated with Lyso Tracker Red and Hoechst for staining intracellular acidic compartments and nuclei, respectively. Scale bars represent 20 μm.

We performed confocal laser scanning microscopic (CLSM) observations of JAWS II cells incubated with various EG*_n_* NFs to evaluate the association of NFs in further detail (Figure 6). The CLSM images of cells incubated with EG_12_ NFs clearly show that the NFs were internalized into cells. The fluorescence signals were observed as dot-like images, indicating that EG_12_ NFs were internalized via endocytosis. In contrast, EG_24_ NFs showed no fluorescence signal, indicating poor cellular uptake of EG_24_ NFs. The confocal images of EG_6_ NFs-treated cells showed large intensive fluorescence signals on the surface of cells, indicating that some aggregation of NFs were apparently adsorbed onto the surface of cells. This observation indicates that a large proportion of the fluorescence signal from EG_6_ NFs-treated cells observed in FCM measurements was derived from NFs that had adhered to the surface of cells. The FCM and CLSM results comprehensively showed the efficient uptake of EG_12_ NFs by cells. 

#### 2.3.2. Cytotoxicity

**Figure 7:**
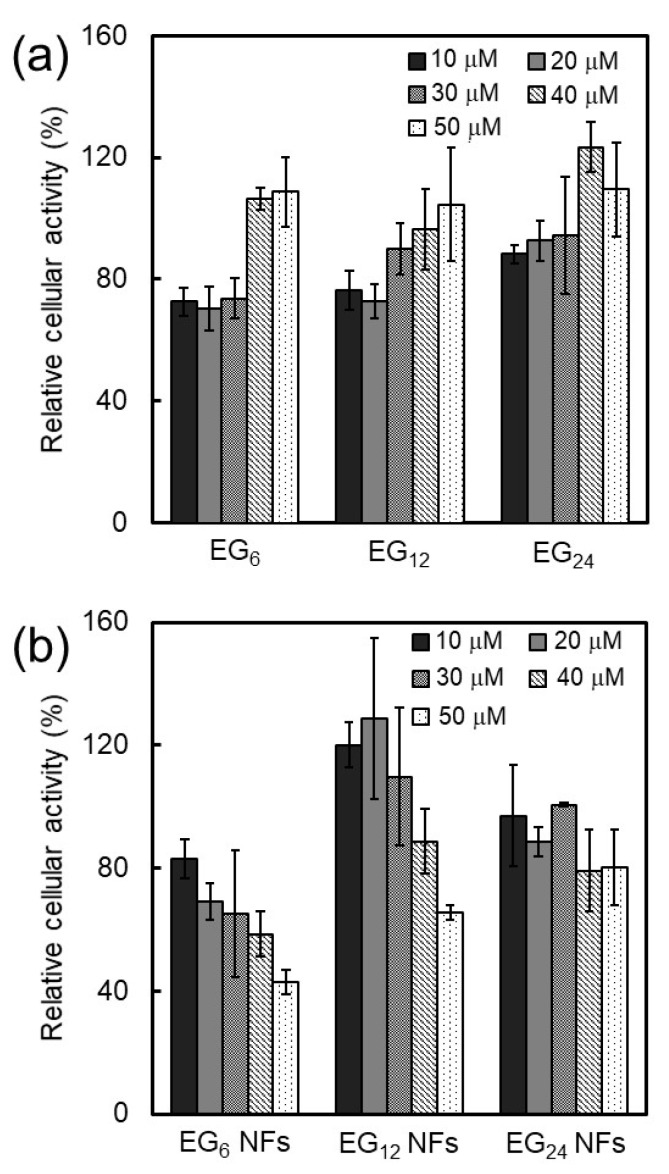
Evaluation of the cytotoxicity of EG*_n_* peptides (**a**) and EG*_n_* NFs (**b**) against JAWS II cells.

The cytotoxicity of EG*_n_* NFs and EG*_n_* peptides (non-fiber) were evaluated. The JAWS II cells were incubated with the EG*_n_* NFs or EG*_n_* peptides at 37 °C for 24 h at different concentrations (10–50 μM), and cell activity was evaluated. Interestingly, cell activity after incubation with all EG*_n_* peptides increased as the peptide concentration increased (Figure 7a). Relative cell activity was essentially 100%, independent of the EG length when peptides were co-incubated with cells at higher concentrations (40–50 μM). Co-incubation of peptides with cells at lower concentrations (10–20 μM) reduced cell activity to 70–90%. In addition, peptides with shorter EG chains showed relatively higher toxicity.

In contrast, cell activity was observed to decrease after incubation with each NF, and this reduction in cell activity was concentration-dependent (Figure 7b). In particular, the activity of EG_6_ NFs-treated cells was reduced to 40% at a concentration of 50 μM. For EG_12_ and EG_24_ NFs, cell activity was 70% for EG_12_ NFs and 80% for EG_24_ NFs even at a NFs concentration of 50 μM. Thus, NFs with shorter EG chains exhibited higher toxicity.

#### 2.3.3. DC Stimulatory Activity.

**Figure 8:**
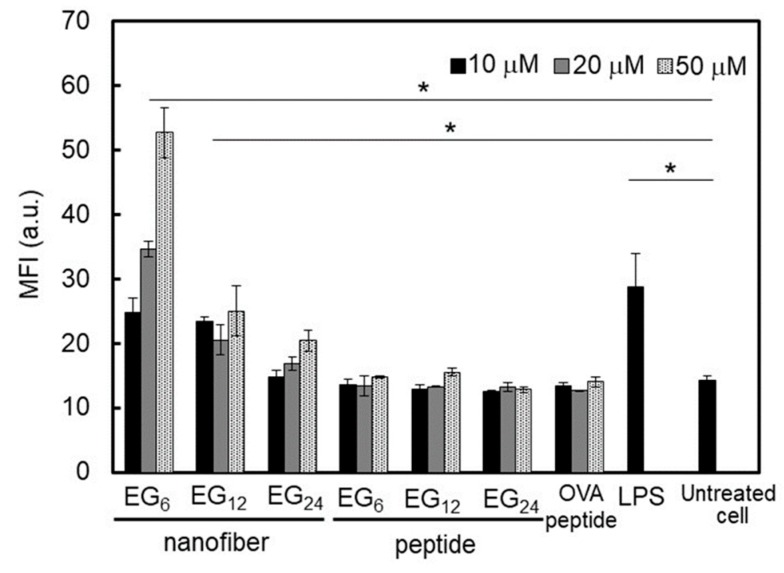
Evaluation of CD86 expressed on the surface of JAWS II cells co-incubated with EG*_n_* NFs (10–50 μM), EG*_n_* peptides (10–50 μM), the OVA peptide (SIINFEKL, 10–50 μM), or 1 μg·mL⁻^1^ LPS at 37 °C for 24 h. CD86 expression was analyzed by FCM. Each result is the mean ± SD (*n* = 3). * *p* < 0.01. a.u. represents arbitrary unit.

It is well known that DC maturation is accompanied by enhanced expression of co-stimulatory molecules (CD40, CD80, and CD86) and by an increase in the secretion of immune-stimulatory cytokines (IL-6, IL-10, IL-12, and TNF-α) [46]. We measured the amount of expressed co-stimulatory molecules (CD86) on JAWS II cells cultured in the presence of EG*_n_* NFs or EG*_n_* peptides for 24 h to determine the effect of EG length on DC maturation. As a positive control, the expression of CD86 on lipopolysaccharides (LPS)-stimulated JAWS II was also measured. The expression of CD86 on JAWS II cells after incubation with EG*_n_* peptides is shown in Figure 8. Even at a peptide concentration of 50 μM, the expression level of CD86 was almost the same as that of untreated JAWS II cells. These results indicate that EG*_n_* peptides do not stimulate DC. In contrast, co-incubation of EG*_n_* NFs with JAWS II cells significantly enhanced the expression of CD86, and this enhancement was dependent on the concentration of the NFs. In particular, when EG_12_ NFs and EG_6_ NFs were co-incubated with JAWS II cells, the amount of expressed CD86 was comparable to or larger than that on LPS-stimulated DC. 

**Figure 9:**
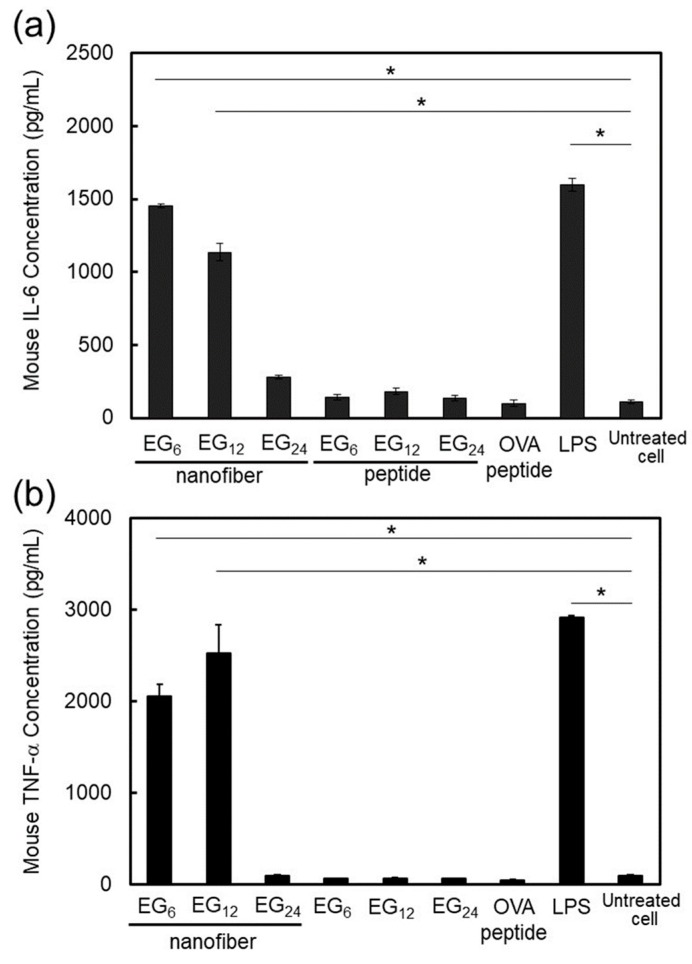
Quantification of immune-stimulatory cytokines secreted from JAWS II cells co-incubated with 10 μM EG*_n_* NFs, 10 μM EG*_n_* peptides, 10 μM OVA peptide (SIINFEKL), or 1 μg·mL⁻^1^ LPS at 37 °C for 24 h. The cytokine levels (**a**,**b**) were measured by ELISA. Each result is the mean ± SD (*n* = 3). * *p* < 0.01.

We also evaluated secretion of immune-stimulatory cytokines. Using enzyme-linked immunosorbent assay (ELISA) methods, we measured the amount of TNF-α and IL-6 contained in the supernatant after 24 h culturing of JAWS II in the presence of EG*_n_* NFs or EG*_n_* peptides (Figure 9). Co-incubation with EG*_n_* peptides did not alter the secretion levels of TNF-α and IL-6. In contrast, interestingly, the secretion of TNF-α and IL-6 was drastically enhanced by co-incubation with EG_6_ NFs and EG_12_ NFs, but not by EG_24_ NFs. These results indicate that NFs with relatively short EG chains have an immune-stimulatory effect as adjuvants for DC maturation.

## 3. Discussion

In this study, we have investigated cellular uptake, cytotoxicity, and DC stimulatory activity of antigen-loaded peptide NFs with different EG lengths and their component peptides. Three building block peptide amphiphiles with different EG lengths (6-mer, 12-mer and 24-mer) were prepared. ThT assay, TEM observation, and CD measurement revealed that all type of peptide amphiphiles are successfully formed β-sheet rich nanofibers with distinct widths (Figure 2, Figure 3 and Figure 4). The association state of EG*_n_* peptides was dependent on sample concentration. EG*_n_* peptides self-assembled into NFs above the CFC, formed spherical micelles at concentrations between the CFC and CAC, and existed as monomers in solution below the CAC. Based on these findings, we discuss separately the effect of EG length on cellular uptake, cytotoxicity, and immune stimulation for three peptide states: NFs, micelles, and monomers. 

### 3.1. Effect of EG Length of Nanofibers on Their Cellular Uptake, Cytotoxicity, and Immune Stimulation Ability

The EG length of NFs significantly affected their cellular uptake, cytotoxicity, and DC stimulatory activity. Here, we discuss the effect of EG length on these properties of NFs using structural models derived from SAXS, WAXD, CD, and FT-IR data of a previous study [36]. FT-IR, CD, and WAXD results indicate that EG_12_ NFs contain β-sheet structures. In addition, synchrotron X-ray scattering profiles of EG_12_ NFs revealed that the morphology of the NFs is rectangular, and they do not form cylinder structures like filament micelles, presumably because of the laminated structure of β-sheets. In general, amyloid-like nanofibers have a common characteristic cross-β-sheet structure, where tightly packed β-sheets orientate themselves perpendicularly to the fiber elongation axis [47]. By combining these findings, we propose a model of EG*_n_* NFs (Figure S7). β-sheet structures consisting mainly of hydrophobic amino acids form the framework of NFs with EG chains facing outwards to provide water-dispersibility. The surfaces of NFs possess hydrophobic and hydrophilic domains that consist of EG chains based on this model. The ANS assay results support the notion that there are hydrophobic domains on the surface of NFs.

#### 3.1.1. Cellular Association and Internalization of NFs

The amount of NFs associated with cells increased in the order of EG_24_ NFs, EG_12_ NFs, and EG_6_ NFs (Figure 5b). Surface hydrophobicity of nanomaterials has been well documented to affect cellular association and uptake by phagocytic cells [24,30,31,32]. Surface hydrophobicity of nanomaterials facilitates interactions between nanomaterial surfaces and cellular membranes. This may lead to higher cell association of nanomaterials and occasionally increase the chance of recognition by particular receptors involved in cellular uptake. Our results show that cellular association of NFs decreased as the EG chain length increased (Figure 5b), although these NFs commonly possess hydrophobic domains on their surface, as evidenced by the ANS fluorescence assay. These results suggest that longer EG chains inhibit hydrophobic interactions between the NF surface and cell membranes, which can be explained using the model structures presented in Figure 9. The NF skeleton region, consisting mainly of hydrophobic amino acids, may facilitate the interaction with the cell membrane and the EG chain located on the lateral face of the NF may inhibit this interaction. 

Results from CLSM observation revealed that EG_12_ NFs were more efficiently internalized by JAWS cells than EG_6_ NFs and EG_24_ NFs (Figure 6). Since the surface of EG_12_ NFs is negatively charged, the mechanism for internalization of EG_12_ NFs would be mainly via phagocytosis by scavenger receptor, which recognizes anion species, although further studies using some inhibitor for phagocytosis are required. Thus, the internalization behavior by non-phagocytic cells would be different from that by JAWS II cells. The internalization of EG_6_ NFs was low, whereas their association propensity to cells was high. Because the size of nanomaterials can affect cell internalization [48,49,50,51], the dispersion state of NFs in aqueous media should be considered in addition to interactions between NFs and the cell surface. The results from DLS indicate that EG_12_ and EG_24_ NFs exist as isolated NFs without aggregation in aqueous media, whereas EG_6_ NFs form large aggregates. This observation is consistent with CLSM images showing large aggregates adsorbed onto the cell surfaces. Thus, it is likely that the low efficiency of cellular internalization of EG_6_ NFs can be attributed to their apparent size in water. The aggregation of EG_6_ NFs is too large for cell uptake. This interpretation is consistent with a previous study that showed that cellular uptake of microparticles with a diameter of a few micrometers or more by phagocytic cells is slow and inefficient [50,51]. Thus, for development of NFs that are efficiently taken up by cells, it is important to design a EG chain length that allows modest interactions with cell membranes while ensuring water-dispersibility. 

#### 3.1.2. Cytotoxicity of NFs

Generally, nanomaterials with cationic or hydrophobic surfaces can induce significantly higher toxicities when compared with hydrophilic or anionic nanomaterials [29]. A mechanism of cytotoxicity is cell membrane perturbation, including structural alternation, pore formation, and phase transitions, which cause nonspecific entrance of extracellular components to the cytosol. An increase in hydrophobic interactions between the surface of nanomaterials and cell membranes could perturb the membrane. In the present study, the cytotoxicity of EG*_n_* NFs was found to increase in the order of EG_24_, EG_12_, and EG_6_ (Figure 7). These results indicate that longer EG chains inhibit the interaction between NFs and cell membranes, which leads to lower cytotoxicity of NFs with long EG chains. It is also possible that the cytotoxicity of NFs may be related to biological stress, e.g., induction of reactive oxygen species (ROS). The detailed mechanism of cytotoxicity by NFs is the subject of ongoing research.

#### 3.1.3. DC Stimulation Ability of NFs

DC activation ability of EG_6_ NFs and EG_12_ NFs was much higher than that of EG_24_ NFs. Matzinger and colleagues have proposed that hydrophobic portions in various biomolecules may be involved in the activation of the immune system [22]. Hydrophobic components in molecules are usually masked from the external environment by hydrophilic components. However, when protein denaturation or cell disruption occur, these hydrophobic components become exposed and interact with particular surface receptors of immune cells, which activates the immune system. In agreement with the notion proposed by Matzinger, recently, the relationship between the surface hydrophobicity of nanomaterials and their immune stimulatory activities has been reported [23,24,25,26,27,28]. For example, Moyano and colleagues reported that the surface hydrophobicity of ligand-modified gold-nanoparticles was correlated with expression of pro-inflammatory cytokine genes in splenocytes from mice in vitro [23]. Shima and colleagues also reported that the activation ability of nanoparticles was significantly affected by the hydrophobicity of polymers constituting the nanoparticles [24]. In the present study, EG_6_ NFs and EG_12_ NFs stimulated DC maturation more effectively than EG_24_ NFs, as evidenced by the quantitative evaluation of expressed co-stimulatory molecules (Figure 8) and secreted immune-stimulatory cytokines, IL-6 and TNF-α (Figure 9). Based on these results, it is reasonable to consider that the hydrophobic part of NFs plays an important role in DC activation. Longer EG chains seem to inhibit the recognition of hydrophobic surfaces of NFs by DC surface receptors in a similar manner to that described above. However the mechanisms responsible for DC maturation by EG*_n_* NFs remain unclear and further studies are required. In addition, because IL-6 signaling cannot only promote anti-tumor-adaptive immunity, but also drive malignancy [52], the role of IL-6 in this NFs-based vaccine system should be examined further in vivo.

### 3.2. Cellular Uptake, Toxicity, and DC Stimulatory Ability of Micelles

In the concentration range where EG*_n_* peptides form micelle-like structures, their cellular association, cytotoxicity, and stimulation ability were not dependent on EG length (Figure 5a, Figure 7a, and Figure 8). These results suggest that the surface components of the micelle-like structures would be almost the same. Poly(ethylene glycol) (PEG) interactions with biological components, including cellular membranes and proteins, are weak because of their nonionic hydrophilicity and high mobility [53]. Thus, a low-level of interaction between the surface of the micelle-like structures and cell membrane components, including receptors involved in cellular uptake and DC maturation, led to lower uptake by DC, lower cytotoxicity, and no DC activation in comparison with NFs. 

### 3.3. Cellular Uptake, Toxicity, and DC Stimulatory Ability of Monomeric Molecules 

EG*_n_* peptides exist as monomeric molecules in aqueous media at concentrations below ca. 15–30 μM. The cellular uptake of monomeric peptides was not dependent on EG length (Figure 5a) and this may be attributed to size. Monomeric peptides are too small for efficient uptake by DC regardless to EG length. In addition, the monomeric peptides exhibited some cytotoxicity but no DC activation ability, which is in sharp contrast to the results with NFs (Figure 7a, Figure 8, and Figure 9). These results suggest that monomeric peptides interact with cell membranes, possibly through an N-terminal hydrophobic region, but are not well recognized by receptors involved in DC activation. Recognition by receptors would be dependent on the size of the hydrophobic portion.

### 3.4. Design of NF-based Vaccines

Recently, various types of NFs for immunotherapy have been reported [54,55]. In particular, NFs formed from antigenic peptides conjugating to β-sheet-forming peptides have been recognized as very promising candidates for next-generation nanoparticle-based vaccines. In the present study, we demonstrated that the hydrophilic-hydrophobic balance of peptide NFs affects their cellular uptake, cytotoxicity, and DC activation ability. NFs consisting of EG with a moderate length (12-mer) showed the most balanced character: Highly efficient cell entry, low cytotoxicity, and high DC activation ability, indicating that the NFs have significant potential as NF-based vaccines, which can be used without additional adjuvants. In general, the relationship between toxicity and DC stimulation ability is a trade-off. It is important to improve the stimulation ability, while simultaneously reducing the cytotoxicity of the NFs. Our results demonstrate that such balance can be simply tuned by the length of the EG. This feature is important for designing safe NF-based vaccines with high immune stimulatory ability. In contrast to NF uptake, the uptake of micelles and monomeric peptides by DC cells inefficient and showed no DC stimulation ability independent of EG length. This result indicates that the assembly style of building block peptide molecules influences the properties of the nanoassembly formed from these building blocks. Finally, to develop NFs with strong immunity-inducing ability, it is necessary to precisely adjust the EG length and introduce intracellular environment-responsive links for efficient release of antigens in cells.

Although we focused on the effect of hydrophilic and hydrophobic balance of nanofibers on their interaction with cells, surface charge of nanofibers is also an important factor in determining the interaction. In general, positively charged nanomaterials are more effectively internalized to cells than negatively charged ones, but they are more toxic. Thus, for design of nanofiber vaccines, it is necessary to address the role of their surface charge. In addition to surface charge, the length of NFs is also an important factor determining their property as a nano-vaccine. In a previous study, we investigated the effect of nanofiber length on their cellular uptake using various NFs with different lengths (40 nm, 120 nm, 280 nm, 800 nm). The study demonstrated that nanofibers with a length of 280 nm were most effectively uptaken by phagocytic cells compared to the others (unpublished data). Based on this finding, we used NFs with a length of 230–260 nm for the cell experiments in the present study. However, other properties—e.g., cytotoxicity and the ability to stimulate immune cells etc.—could exhibit different size-dependencies. Therefore, the optimization of nanofiber length is also required for developing effective nanofiber vaccine.

The important attributes of a vaccine, which are antigen processing, antigen presentation, T-cell stimulation, and successful activation of adaptive immune response against target antigen, should also be evaluated. However, because the NFs used in this study comprised the minimum required block (β-sheet forming peptide, antigenic peptide, oligo(ethylene glycol)) and the antigen could not be released in the cells, the effective antigen presentation via MHC class I pathway and subsequent induction of immunity are not expected. Therefore, we are addressing the development of intracellular environment-responsive NFs for efficient release of antigens in cells and the characterization of their function to induce immunity in vivo.

## 4. Materials and Methods

### 4.1. Materials

21-amino-*N*-(9-fluorenylmethoxycarbonyl)-4,7,10,13,16,19-hexaoxaheneicosanoic acid (Fmoc-*N*-amido-dPEG_6_ acid), 39-amino-*N*-(9-fluorenylmethoxycarbonyl)-4, 7, 10, 13, 16, 19, 22, 25, 28, 31, 34, 37-dodecaoxanonatriacontanoic acid (Fmoc-*N*-amido-dPEG_12_ acid), and O-[*N*-(9-fluorenylmethoxycarbonyl)-2-aminoethyl]-O’-(2-carboxyethyl) undecaethyleneglycol (Fmoc-*N*-amido-dPEG_24_ acid) were purchased from Quanta BioDesign Ltd. (Plain City, OH, USA). 2-chlorotrityl chloride resin, *N*,*N*’-diisopropylethylamine (DIPEA), all the L-Fmoc amino acids, 1-[bis(dimethylamino)methylene]-1H-benzotriazolium 3-oxide hexafluorophosphate (HBTU), 1-hydroxybenzotriazole (HOBT), and piperidine were purchased from Watanabe Chemical Industries Ltd. (Hiroshima, Japan). *N*,*N*’-dimethylformamide (DMF), isopropanol, methanol, diethyl ether, hexafluoroisopropanol (HFIP), dichloromethane (CH_2_Cl_2_), trifluoroacetic acid (TFA), and granulocyte-macrophage colony-stimulating factor (GM-CSF) were purchased from Wako Pure Chemical Industries, Ltd. (Osaka, Japan). Eagle’s minimal essential medium (EMEM), penicillin-streptomycin, and lipopolysaccharides from *Escherichia coli* O55:B5 were purchased from Sigma-Aldrich (St. Louis, MO, USA). *N*^6^-[(3′,6′-Dihydroxy-3-oxospiro[isobenzofuran-1(*3H*),9′-[*9H*]xanthen]-5-yl)carbonyl]-*N*^2^-[(*9H*-fluoren-9-ylmethoxy)carbonyl]-L-lysine (Fmoc-Lys(5-FAM)-OH) was purchased from AAT Bioquest (Sunnyvale, CA, USA). LysoTracker Red DND-99, and Hoechst 33342, trihydrochloride, trihydrate were purchased from Thermo Fisher Scientific (Waltham, MA, USA). Fetal bovine serum (FBS) was purchased from Biowest (Nuaillé, France). Purified anti-mouse CD16/CD32 (2.4G2) and anti-mouse CD86 (B7-2) PE were purchased from Tonbo Biosciences (San Diego, CA, USA). The ELISA Kit for mouse TNF-alpha and IL-6 were purchased from R&D Systems (Minneapolis, MN, USA).

### 4.2. Experimental Methods

#### 4.2.1. Synthesis of Building Block Molecules 

Loading of resin: Fmoc-*N*-amido-dPEG_12_ was dehydrated by azeotropy with benzene prior to use. A solution of Fmoc-*N*-amido-dPEG_12_ acid (0.238 mmol) and DIPEA (0.952 mmol) in CH_2_Cl_2_ (2.6 mL) was added to 2-chlorotrityl chloride resin (0.397 mmol, 1.5 mmol/g loading max) for 12 h.

Peptide synthesis: Coupling reactions were performed using a standard Fmoc protocol. The coupling cycle included 3 repeats of Fmoc deprotection (20% piperidine in DMF, 5 min), a wash in DMF, two repeats of amino acid coupling: L-Fmoc amino acids (4 eq.), HBTU (3.6 eq.), HOBt (4 eq.), and DIPEA (8 eq.) for 30 min, and a final DMF wash. After all coupling reactions, the obtained peptides were cleaved from the resin using a solution of H_2_O/TFA/triisopropylsilane (100:5:2 volume ratio) containing 500 mM phenol for 2 h. The resulting peptides were precipitated in ice cold diethyl ether, filtered, centrifuged, and washed with diethyl ether. The crude peptides were purified by reversed-phase high-performance liquid chromatography (RP-HPLC, SPD-10A and LC-10A, Shimadzu Scientific Instruments, Kyoto, Japan). Other building blocks with different EG lengths were synthesized by a similar procedure. Molecular weight was analyzed by MALDI-TOF mass (autoflex speed system, Bruker, Billerica, MA, USA). MS (MALDI-TOF): EG_6_; Cald. MASS: 2234.92, Obsd. MASS: 2234.359, EG_12_; Cald. MASS: 2499.12, Obsd. MASS: 2498.11, EG_24_; Cald. MASS: 3027.52, Obsd. MASS: 3027.04.

Fluorescence-labeled building block peptides were synthesized using Fmoc-Lys(5-FAM)-OH as the first amino acid residue by a similar procedure. MS (MALDI-TOF): EG_6_-FAM; Cald. MASS: 2721.41, Obsd. MASS: 2720.84, EG_12_-FAM; Cald. MASS: 2985.61, Obsd. MASS: 2985.26, EG_24_-FAM; Cald. MASS: 3514.01, Obsd. MASS: 3514.65.

#### 4.2.2. Preparation of Antigen-Loaded Peptide NFs

EG*_n_* peptide was dissolved in HFIP and dried with nitrogen flow to allow film formation. The obtained film was re-dissolved at a concentration of 30 mM in DMSO. The resulting solution (15 μL) was added to PBS (285 μL) to a final concentration of 1.5 mM and incubated at 60 °C for 24 h. Following incubation, the resulting peptide nanofiber dispersion was dialyzed against PBS for 24 h using dialysis membrane (MWCO 8,000, GE Healthcare, Chicago, IL, USA) to remove DMSO and free peptides. For cell-based experiments (cytotoxicity, DC maturation), the length of NFs was controlled by filtration using a syringe filter with a pore size of 0.45 μm (GE Healthcare).

#### 4.2.3. Preparation of Fluorescence-Labeled Antigen-Loaded Peptide NFs

The HFIP-treated mixture of EG*_n_* and EG*_n_*-FAM peptides was dissolved in DMSO; then, 15 μL of the solution was added to 285 μL PBS to give final concentrations of 1.425 mM for EG*_n_* and 0.071 mM for EG*_n_*-FAM. The solution was incubated at 60 °C for 24 h, and then the resulting peptide nanofiber dispersion was dialyzed against PBS for 24 h using dialysis membrane (MWCO 8,000, GE Healthcare) to remove DMSO and free peptides. The length of NFs was controlled by filtration using a syringe filter with a pore size of 0.45 μm (GE Healthcare).

#### 4.2.4. Determination of Critical Aggregation Concentration 

CAC was determined using the pyrene 1:3 method [56]. First, a saturated solution of pyrene was prepared by mixing an excess of pyrene with PBS, and using the supernatant to dissolve EG*_n_* peptides at a concentration of 150 mM (the stock solution). A concentration range of EG*_n_* peptides from 2.5 μM to 100 μM was then prepared using serial dilutions of the stock solution with the saturated solution of pyrene. The final concentration of pyrene was equal in each solution. The fluorescence emission of pyrene was monitored using a fluorescence spectrometer (RF5300 PC, Shimadzu Scientific Instruments, Kyoto, Japan) with an excitation wavelength of 335 nm at 37 °C. The ratio of the emission intensities at 376 nm and 392 nm were then plotted as a function of the EG*_n_* peptide concentration (log scale). The CAC was determined from an abrupt change in the slope of the plot using the least-squares fitting technique.

#### 4.2.5. ThT Assay

PBS containing 10 μM Thioflavin T (ThT) was dispensed into a 96-well plate. EG*_n_* peptides solution (6 mM) was prepared and added to the 96-well plate, giving a final concentration of 300 μM. ThT fluorescence intensities at 480 nm (excitation; 440 nm) were monitored using a Genios microplate reader (TECAN, Männedorf, Switzerland) at 37 °C.

#### 4.2.6. Measurement of Surface Hydrophobicity

The surface hydrophobicity of NFs in the solution was determined using an ANS fluorescent probe as previously reported [41,42]. A concentration range of EG*_n_* NFs in PBS from 9.4 μM to 200 μM was prepared. The nanofiber dispersion was mixed with the equivalent volume of PBS containing 20 μM ANS. The intensities of ANS fluorescence ranging from 400 nm to 600 nm (excitation; 370 nm) were monitored using a fluorescence spectrometer (RF5300 PC) at 37 °C.

#### 4.2.7. Cell Culture

JAWS II, a DC line derived from mouse bone marrow, was purchased from the American Type Culture Collection (ATCC, Manassas, VA, USA). The cells were grown in EMEM supplemented with 20% FBS, 5 ng/mL murine GM-CSF, and antibiotics at 37 °C, 5% CO_2_.

#### 4.2.8. Evaluation of Cellular Association of Peptide NFs

JAWS II cells were seeded into 12-well plates (2.5 × 10^5^ per well) and cultured for 12 h at 37 °C in a humidified atmosphere (5% CO_2_). After 12 h, the cells were washed with PBS and serum-free culture medium. The fluorescence-labeled peptide NF dispersion was added gently to the cells followed by incubation for 2 h at 37 °C. Following incubation, the cells were washed with PBS and 0.2% trypan blue aqueous solution, which was used to quench the flourescence from surface-adsorbed NFs [57]. The cells were then detached using trypsin and subsequently analyzed by FCM (Guava EasyCyte Plus, Millipore, Burlington, MA, USA). As a comparison to peptide NFs, the cellular uptake of the building block peptides without heat treatment was investigated under the same conditions.

#### 4.2.9. CLSM Observation of NF-Treated Cells

JAWS II cells (1.5 × 10^5^) were cultured for 12 h in a 35 mm glass-bottom dish and subsequently washed with PBS and serum-free culture medium. Fluorescein-labeled peptide NFs were gently added to the cells, followed by incubation for 2 h at 37 °C with 5% CO_2_. After incubation, the cells were washed with PBS, and then incubated for 5 min with a solution containing LysoTracker Red DND-99 (50 nM) and Hoechst 33342, trihydrochloride, trihydrate (3.24 μM). LysoTracker Red DND-99 and Hoechst 33342 were used to stain the intracellular acidic compartments and nuclei, respectively. After staining, the cells were washed twice with PBS, then observed by CLSM using an FV10i microscope (Olympus, Tokyo, Japan). 

#### 4.2.10. Quantitative Expression Analysis of Co-Stimulatory Molecules and Cytokines from NF-Treated Cells

The expression of co-stimulatory molecules and cytokines was evaluated by specific immunostaining, as well as by ELISA. For immunostaining, JAWS II cells (2 × 10^5^) were cultured for 12 h in a 24-well plate followed by washing with PBS containing 3% FBS and 0.05% NaN_3_, and then with serum-free culture medium. The DCs were pulsed with peptide NFs for 24 h, and then immunostained with a mouse monoclonal antibody for CD86 (a maturation marker), and subsequently analyzed by FCM to estimate their CD86 expression level. For quantitative analysis of TNF-α and IL-6 expression, the supernatants following the 24 h co-incubation of DCs with peptide NFs were collected and analyzed using an ELISA kit. The maturation of JAWS II cells cultured in medium with and without LPS (1 μg/mL) was evaluated as the positive and negative control, respectively. In addition, to compare the DC-activation ability between the NFs and heat-untreated building block peptides, JAWS II cells cultured with the peptides were also evaluated. 

#### 4.2.11. Evaluation of Cytotoxicity of Peptide NFs

The cytotoxicity of peptide NFs was evaluated using a Cell Counting Kit-8 (Dojindo Molecular Technologies, Kumamoto, Japan) according to the manufacturer’s instructions. Briefly, JAWS II cells were seeded into 96-well plates (1.0 × 10^5^ per well) and cultured for 12 h at 37 °C in a humidified atmosphere (5% CO_2_). After 12 h, the cells were washed with PBS and serum-free culture medium. The nanofiber dispersion was gently added to the cells followed by incubation for 24 h. The cells were washed with PBS three times and the medium was replaced with a solution containing 2-(2-methoxy-4-nitrophenyl)-3-(4-nitrophenyl)-5-(2,4-disulfophenyl)-2H-tetrazolium, monosodium salt (WST-8), and 1-methoxy-5-methylphenazinium methylsulfate at a 10-fold dilution. After a 2 h incubation, the absorbance was measured at 420 nm using a plate reader (Multiskan JX, Thermo Fisher Scientific, Waltham, MA, USA). The relative cellular activity was calculated using the following equation:

(1) $$\%\;\mathrm{relative}\;\mathrm{cellular}\;\mathrm{activity}=\frac{A_{420\;\mathrm{nm}}\;\left(\mathrm{NFs}-\mathrm{treated}\;\mathrm{cells}\right)-A_{420\;\mathrm{nm}}\;\left(\mathrm{blank}\right)}{A_{420\;\mathrm{nm}}\left(\mathrm{untreated}\;\mathrm{cells}\right)-A_{420\;\mathrm{nm}}\;\left(\mathrm{blank}\right)}\times 100$$

where *A*_420 nm_ is the absorbance at 420 nm, *A*_420 nm_ (untreated cells) is the absorbance at 420 nm after incubation in the absence of peptide NFs, and *A*_420 nm_ (blank) is the absorbance of medium containing WST-8 reagent at 420 nm. As a comparison, the cytotoxicity of building block peptides without heat treatment was investigated in a similar manner.

### 4.3. Other Characterizations

TEM measurements were performed using a JEM-1200EX II (JEOL, Tokyo, Japan) with an acceleration voltage of 85 keV. The samples were negatively stained with 0.1% phosphotungstate. p-potentials of NFs were measured using a Micro-Electrophoresis Zeta Potential Analyzer Model 502 (Nihon Rufuto, Tokyo, Japan). DLS analysis was performed using a particle size analyzer (ELSZ-1000, Otsuka Electronics, Osaka, Japan) at 25 °C. The light source was a He-Ne laser (630 nm) set at an 1ngle of 45°. Experimental data were analyzed using the marquardt provided by the manufacturer. CD spectra were measured using a J-720 spectropolarimeter (Jasco, Tokyo, Japan) at 25 °C. The data were obtained using a 0.1 cm path length cell at a scan speed of 20 nm/min.

## 5. Conclusions

This study showed that the hydrophilic-hydrophobic balance of antigen-loaded NFs significantly impacted on their cellular uptake, cytotoxicity, and DC stimulation ability, which differs noticeably from the results observed for micelles formed from the same components of NFs. Building blocks consisting of β-sheet-forming peptides conjugated with antigenic peptides and hydrophilic EG with different lengths (6-mer, 12-mer and 24-mer) were found to successfully form NFs with homogenous widths. The uptake of NFs consisting of EG with a moderate length (12-mer) by DC was effective, and these NFs activated DC without exhibiting significant cytotoxicity. Increasing the EG chain length significantly reduced the interactions with cells. Conversely, decreasing the EG chain length enhanced DC activation ability but increased toxicity and impaired water-dispersibility, resulting in low cellular uptake. Thus, since cell entry, cytotoxicity, and the immune stimulation ability of antigen-loaded NFs can be tuned by the length of the EG moiety, the antigen-loaded NFs have potential as NF-based vaccines that can be used without additional adjuvants. In order to achieve efficient immune response in vivo, the development of intracellular environment-responsive NFs is now in progress. We believe the findings obtained in this study contribute to the understanding of the interaction between the surface of one-dimensional assemblies and cells, and provide useful design guidelines for development of effective NF-based vaccines.

## Supplementary Materials

Supplementary materials can be found at https://www.mdpi.com/1422-0067/20/15/3781/s1.

## References

[1] Skwarczynski M., Toth I. (2016). Peptide-based synthetic vaccines. Chem. Sci..

[2] Li W., Joshi M., Singhania S., Ramsey K., Murthy A. (2014). Peptide vaccine: Progress and challenges. Vaccines.

[3] Yoshizaki Y., Yuba E., Komatsu T., Udaka K., Harada A., Kono K. (2016). Improvement of peptide-based tumor immunotherapy using pH-sensitive fusogenic polymer-modified liposomes. Molecules.

[4] Varypataki E.M., Silva A.L., Barnier-Quer C., Collin N., Ossendorp F., Jiskoot W. (2016). Synthetic long peptide-based vaccine formulations for induction of cell mediated immunity: A comparative study of cationic liposomes and PLGA nanoparticles. J. Control. Release.

[5] Guan H.H., Budzynski W., Koganty R.R., Krantz M.J., Reddish M.A., Rogers J.A., Longenecker B.M., Samuel J. (1998). Liposomal formulations of synthetic MUC1 peptides: Effects of encapsulation versus surface display of peptides on immune responses. Bioconjugate Chem..

[6] Büyüktimkin B., Wang Q., Kiptoo P., Stewart J.M., Berkland C., Siahaan T.J. (2012). Vaccine-like controlled-release delivery of an immunomodulating peptide to treat experimental autoimmune encephalomyelitis. Mol. Pharm..

[7] Chua B.Y., Al Kobaisi M., Zeng W., Mainwaring D., Jackson D.C. (2011). Chitosan microparticles and nanoparticles as biocompatible delivery vehicles for peptide and protein-based immunocontraceptive vaccines. Mol. Pharm..

[8] Ma W., Chen M., Kaushal S., McElroy M., Zhang Y., Ozkan C., Bouvet M., Kruse C., Grotjahn D., Ichum T. (2012). PLGA nanoparticle-mediated delivery of tumor antigenic peptides elicits effective immune responses. Int. J. Nanomed..

[9] Zeng Q., Jiang H., Wang T., Zhang Z., Gong T., Sun X. (2015). Cationic micelle delivery of Trp2 peptide for efficient lymphatic draining and enhanced cytotoxic T-lymphocyte responses. J. Control. Release.

[10] Zhao G., Chandrudu S., Skwarczynski M., Toth I. (2017). The application of self-assembled nanostructures in peptide-based subunit vaccine development. Eur. Polym. J..

[11] Wen Y., Collier J.H. (2015). Supramolecular peptide vaccines: Tuning adaptive immunity. Curr. Opin. Immunol..

[12] Black M., Trent A., Kostenko Y., Lee J.S., Olive C., Tirrell M. (2012). Self-assembled peptide amphiphile micelles containing a cytotoxic T-Cell epitope promote a protective immune response in vivo. Adv. Mater..

[13] Ghasparian A., Riedel T., Koomullil J., Moehle K., Gorba C., Svergun D.I., Perriman A.W., Mann S., Tamborrini M.,  Pluschke G. (2012). Engineered Synthetic Virus-Like Particles and Their Use in Vaccine Delivery. ChemBioChem.

[14] Simerska P., Suksamran T., Ziora Z.M., de Labastida Rivera F., Engwerda C., Toth I. (2014). Ovalbumin lipid core peptide vaccines and their CD4+ and CD8+ T cell responses. Vaccine.

[15] Kakwere H., Ingham E.S., Allen R., Mahakian L.M., Tam S.M., Zhang H., Silvestrini M.T., Lewis J.S., Ferrara K.W. (2017). Toward personalized peptide-based cancer nanovaccines: A facile and versatile synthetic approach. Bioconjugate Chem..

[16] Skwarczynski M., Zaman M., Urbani C.N., Lin I.C., Jia Z., Batzloff M.R., Good M.F., Monteiro M.J., Toth I. (2010). Polyacrylate dendrimer nanoparticles: A self-adjuvanting vaccine delivery system. Angew. Chem. Int. Ed..

[17] Rudra J.S., Tian Y.F., Jung J.P., Collier J.H. (2010). A self-assembling peptide acting as an immune adjuvant. Proc. Natl. Acad. Sci. USA.

[18] Rudra J.S., Mishra S., Chong A.S., Mitchell R.A., Nardin E.H., Nussenzweig V., Collier J.H. (2012). Self-assembled peptide nanofibers raising durable antibody responses against a malaria epitope. Biomaterials.

[19] Pompano R.R., Chen J., Verbus E.A., Han H., Fridman A., McNeely T., Collier J.H., Chong A.S. (2014). Titrating T-Cell Epitopes within Self-Assembled Vaccines Optimizes CD4+ Helper T Cell and Antibody Outputs. Adv. Health Mater..

[20] Huang Z.H., Shi L., Ma J.W., Sun Z.Y., Cai H., Chen Y.X., Zhao Y.F., Li Y.M. (2012). A totally synthetic, self-assembling, adjuvant-free MUC1 glycopeptide vaccine for cancer therapy. J. Am. Chem. Soc..

[21] Si Y., Wen Y., Kelly S.H., Chong A.S., Collier J.H. (2018). Intranasal delivery of adjuvant-free peptide nanofibers elicits resident CD8+ T cell responses. J. Control. Release.

[22] Seong S.Y., Matzinger P. (2004). Hydrophobicity: An ancient damage-associated molecular pattern that initiates innate immune responses. Nat. Rev. Immunol..

[23] Moyano D.F., Goldsmith M., Solfiell D.J., Landesman-Milo D., Miranda O.R., Peer D., Rotello V.M. (2012). Nanoparticle hydrophobicity dictates immune response. J. Am. Chem. Soc..

[24] Shima F., Akagi T., Akashi M. (2015). Effect of hydrophobic side chains in the induction of immune responses by nanoparticle adjuvants consisting of amphiphilic poly (γ-glutamic acid). Bioconjugate Chem..

[25] Liu Y., Yin Y., Wang L., Zhang W., Chen X., Yang X., Xu J., Ma G. (2013). Surface hydrophobicity of microparticles modulates adjuvanticity. J. Mater. Chem. B.

[26] Shahbazi M.A., Fernández T.D., Mäkilä E.M., Le Guével X., Mayorga C., Kaasalainen M.H., Salonen J.J., Hivonen J.T., Santos H.A. (2014). Surface chemistry dependent immunostimulative potential of porous silicon nanoplatforms. Biomaterials.

[27] Moyano D.F., Liu Y., Peer D., Rotello V.M. (2016). Modulation of immune response using engineered nanoparticle surfaces. Small.

[28] Gause K.T., Wheatley A.K., Cui J., Yan Y., Kent S.J., Caruso F. (2017). Immunological principles guiding the rational design of particles for vaccine delivery. ACS Nano.

[29] Saei A.A., Yazdani M., Lohse S.E., Bakhtiary Z., Serpooshan V., Ghavami M., Asadian M., Mashaghi S., Dreaden E.C., Mashaghi A. (2017). Nanoparticle surface functionality dictates cellular and systemic toxicity. Chem. Mater..

[30] Torres M.P., Wilson-Welder J.H., Lopac S.K., Phanse Y., Carrillo-Conde B., Ramer-Tait A.E., Bellaire B.H., Wannemuehler M.J., Narasimhan B. (2011). Polyanhydride microparticles enhance dendritic cell antigen presentation and activation. Acta Biomater..

[31] Ulery B.D., Phanse Y., Sinha A., Wannemuehler M.J., Narasimhan B., Bellaire B.H. (2009). Polymer chemistry influences monocytic uptake of polyanhydride nanospheres. Pharm. Res..

[32] Chiu Y.L., Ho Y.C., Chen Y.M., Peng S.F., Ke C.J., Chen K.J., Mi F.L., Sung H.W. (2010). The characteristics, cellular uptake and intracellular trafficking of nanoparticles made of hydrophobically-modified chitosan. J. Control. Release.

[33] Fukuhara S., Nishigaki T., Miyata K., Tsuchiya N., Waku T., Tanaka N. (2012). Mechanism of the chaperone-like and antichaperone activities of amyloid fibrils of peptides from αA-crystallin. Biochemistry.

[34] Tanaka N., Tanaka R., Tokuhara M., Kunugi S., Lee Y.F., Hamada D. (2008). Amyloid fibril formation and chaperone-like activity of peptides from αA-crystallin. Biochemistry.

[35] Waku T., Kitagawa Y., Kawabata K., Nishigaki S., Kunugi S., Tanaka N. (2013). Self-assembled β-sheet peptide nanofibers for efficient antigen delivery. Chem. Lett..

[36] Minami T., Matsumoto S., Sanada Y., Waku T., Tanaka N., Sakurai K. (2016). Rod-like architecture and cross-sectional structure of an amyloid protofilament-like peptide supermolecule in aqueous solution. Polym. J..

[37] Waku T., Tanaka N. (2017). Recent advances in nanofibrous assemblies based on β-sheet-forming peptides for biomedical applications. Polym. Int..

[38] Inoue M., Konno T., Tainaka K., Nakata E., Yoshida H.O., Morii T. (2012). Positional effects of phosphorylation on the stability and morphology of tau-related amyloid fibrils. Biochemistry.

[39] Lomakin A., Chung D.S., Benedek G.B., Kirschner D.A., Teplow D.B. (1996). On the nucleation and growth of amyloid beta-protein fibrils: Detection of nuclei and quantitation of rate constants. Proc. Natl. Acad. Sci. USA.

[40] Ghosh A., Haverick M., Stump K., Yang X., Tweedle M.F., Goldberger J.E. (2012). Fine-tuning the pH trigger of self-assembly. J. Am. Chem. Soc..

[41] Semisotnov G.V., Rodionova N.V., Razgulyaev O.I., Uversky V.N., Gripas A.F., Gilmanshin R.I. (1991). Study of the molten globule intermediate state by hydrophobic fluorescent probe. Biopolymers.

[42] Yamaguchi S., Mannen T., Nagamune T. (2004). Evaluation of surface hydrophobicity of immobilized protein with a surface plasmon resonance sensor. Biotechnol. Lett..

[43] Greenfield N.J., Fasman G.D. (1969). Computed circular dichroism spectra for the evaluation of protein conformation. Biochemistry.

[44] Shaheen S.M., Akita H., Nakamura T., Takayama S., Futaki S., Yamashita A., Katoono R., Yui N., Harashima H. (2011). KALA-modified multi-layered nanoparticles as gene carriers for MHC class-I mediated antigen presentation for a DNA vaccine. Biomaterials.

[45] Jiang X., Shen C., Rey-Ladino J., Yu H., Brunham R.C. (2008). Characterization of murine dendritic cell line JAWS II and primary bone marrow-derived dendritic cells in Chlamydia muridarum antigen presentation and induction of protective immunity. Infect. Immun..

[46] Yi A.K., Yoon J.G., Hong S.C., Redford T.W., Krieg A.M. (2001). Lipopolysaccharide and CpG DNA synergize for tumor necrosis factor-α production through activation of NF-κB. Int. Immunol..

[47] Kirschner D.A., Abraham C., Selkoe D.J. (1986). X-ray diffraction from intraneuronal paired helical filaments and extraneuronal amyloid fibers in Alzheimer disease indicates cross-beta conformation. Proc. Natl. Acad. Sci. USA.

[48] Shang L., Nienhaus K., Nienhaus G.U. (2014). Engineered nanoparticles interacting with cells: Size matters. J. Nanobiotechnol..

[49] Foged C., Brodin B., Frokjaer S., Sundblad A. (2005). Particle size and surface charge affect particle uptake by human dendritic cells in an in vitro model. Int. J. Pharm..

[50] Tabata Y., Ikada Y. (1988). Effect of the size and surface charge of polymer microspheres on their phagocytosis by macrophage. Biomaterials.

[51] Ayhan H., Tuncel A., Bor N., Pişkin E. (1996). Phagocytosis of monosize polystyrene-based microspheres having different size and surface properties. J. Biomater. Sci. Polym. Ed..

[52] Fisher D.T., Appenheimer M.M., Evans S.S. (2014). The two faces of IL-6 in the tumor microenvironmnt. Semin. Immunol..

[53] Zheng M., Davidson F., Huang X. (2003). Ethylene glycol monolayer protected nanoparticles for eliminating nonspecific binding with biological molecules. J. Am. Chem. Soc..

[54] Xing R., Li S., Zhang N., Shen G., Möhwald H., Yan X. (2017). Self-Assembled Injectable Peptide Hydrogels Capable of Triggering Antitumor Immune Response. Biomacromolecules.

[55] Sirc J., Hampejsova Z., Trnovska J., Kozlik P., Hrib J., Hobzova R., Zajicova A., Holan V., Bosakova Z. (2017). Cyclosporine A Loaded Electrospun Poly(D,L-Lactic Acid)/Poly(Ethylene Glycol) Nanofibers: Drug Carriers Utilizable in Local Immunosuppression. Pharm. Res..

[56] Meng Q., Kou Y., Ma X., Liang Y., Guo L., Ni C., Liu K. (2012). Tunable self-assembled peptide amphiphile nanostructures. Langmuir.

[57] Gratton S.E., Ropp P.A., Pohlhaus P.D., Luft J.C., Madden V.J., Napier M.E., DeSimone J.M. (2008). The effect of particle design on cellular internalization pathways. Proc. Natl. Acad. Sci. USA.

