# Supplementary material for: Effect of the Hydrophilic-Hydrophobic Balance of Antigen-Loaded Peptide Nanofibers on Their Cellular Uptake, Cellular Toxicity, and Immune Stimulatory Properties
Source: Int J Mol Sci. 2019 Aug 2;20(15):3781. doi: 10.3390/ijms20153781 (PMC6696487; doi:10.3390/ijms20153781)
Supplement: Supplementary file 1 [file ijms-20-03781-s001.pdf]

*Supporting Information*

# Effect of the hydrophilic-hydrophobic balance of antigen-loaded peptide nanofibers on their cellular uptake, cellular toxicity and immune stimulatory properties

*Tomonori Waku [1,*], Saki Nishigaki [1], Yuichi Kitagawa [1], Sayaka Koeda [1], Kazufumi Kawabata [1], Shigeru Kunugi [1], Akio Kobori [1], Naoki Tanaka [1]*

[1] Faculty of Molecular Chemistry and Engineering, Kyoto Institute of Technology, Gosyokaido-cho, Matsugasaki, Sakyo-ku, Kyoto 606-8585, Japan

*To whom correspondence should be addressed.

Faculty of Molecular Chemistry and Engineering, Kyoto Institute of Technology, Gosyokaido-cho, Matsugasaki, Sakyo-ku, Kyoto 606-8585, Japan. Tel.: +81 75 724 7811; Fax: +81 75 724 7861; E-mail: waku1214@kit.ac.jp

**Contents**

**Figure S1.** Time-dependent change in the ThT fluorescence intensity of solutions containing the (a) $EG_6$ peptide, (b) $EG_{12}$ peptide and (c) $EG_{24}$ peptide when incubated at 37 °C.

**Figure S2.** Plots of the pyrene I1/I3 ratio versus concentration for the (a) $EG_6$ peptide, (b) $EG_{12}$ peptide and (c) $EG_{24}$ peptide.

**Figure S3.** (a-c) Fluorescence spectra of ANS in the presence of (a) $EG_6$ NFs, (b) $EG_{12}$ NFs and (c) $EG_{24}$ NFs at concentrations of 10 μM in PBS. The concentration of the nanofibers ranged from 0 to 100 μM. (d) Shift in the wavelength peak maximum in fluorescence spectra of ANS (10 μM) in the presence of $EG_6$ NFs (circle), $EG_{12}$ NFs (square) and $EG_{24}$ NFs (diamond) at different peptide concentrations.

**Figure S4.** Negatively stained TEM images of short $EG_n$ NFs obtained by extrusion of nanofiber dispersions through a membrane filter with a pore diameter of 0.45 μm. (a) $EG_6$ NFs, (b) $EG_{12}$ NFs and (c) $EG_{24}$ NFs.

**Figure S5.** Size distribution of short $EG_n$ NFs estimated from TEM images shown in Figure S3. (a) $EG_6$ NFs, (b) $EG_{12}$ NFs and (c) $EG_{24}$ NFs.

**Figure S6.** Size distribution of short $EG_n$ NFs in PBS measured by DLS. (a) $EG_6$ NFs, (b) $EG_{12}$ NFs and (c) $EG_{24}$ NFs.

**Figure S7.** Schematic illustration of the interactions between $EG_n$ NFs and the cellular surface. Double head arrows represent the interactions and the width means the degree of them. Longer PEG chains inhibit interaction with cells, leading to low cellular uptake efficiency, low cytotoxicity and no immune-stimulation ability.

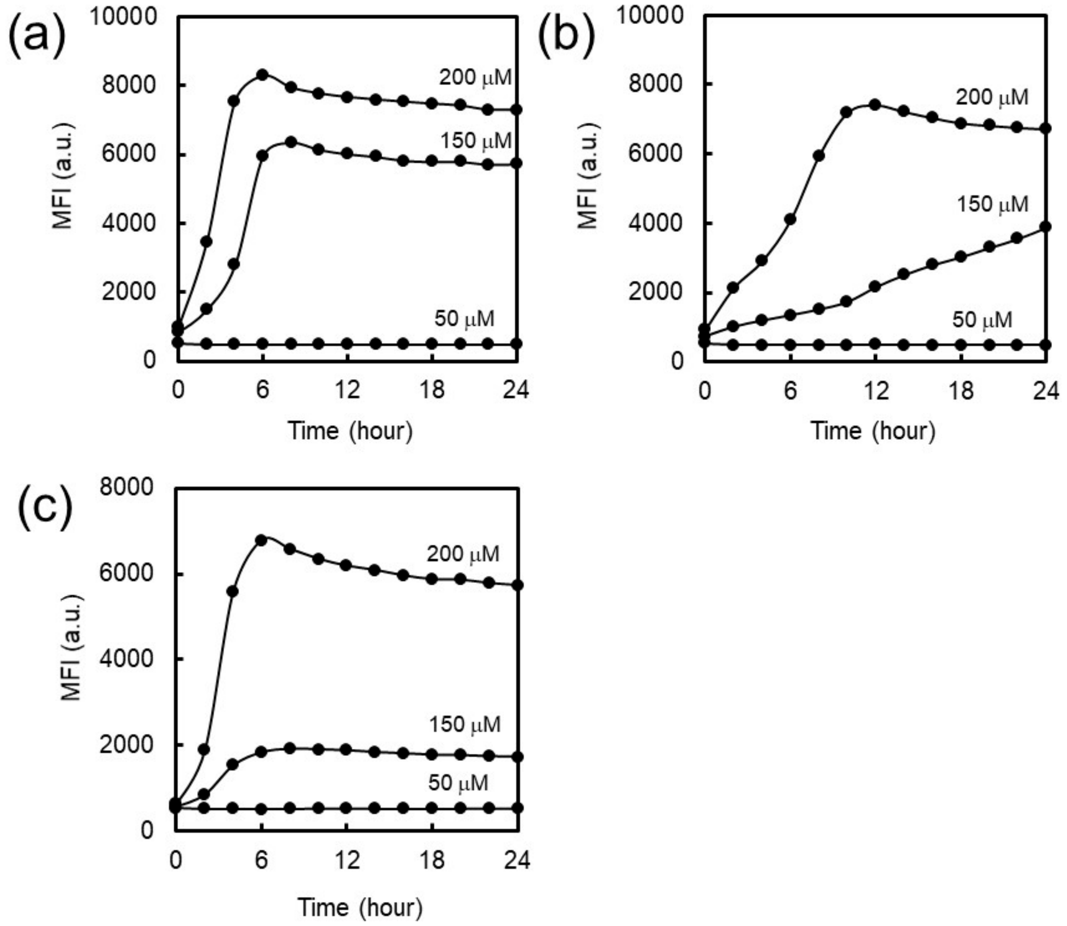


**Figure S1.** Time-dependent change in the ThT fluorescence intensity of solutions containing the (a) $EG_6$ peptide, (b) $EG_{12}$ peptide and (c) $EG_{24}$ peptide when incubated at 37 °C.

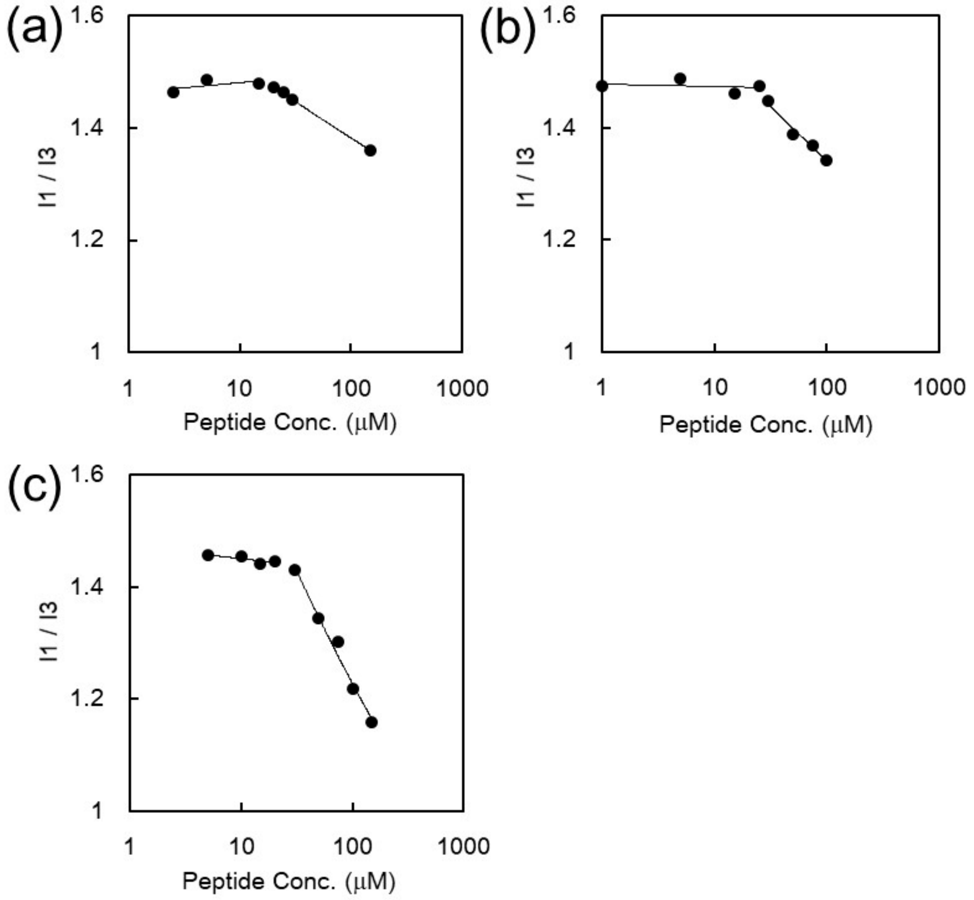


**Figure S2.** Plots of the pyrene I1/I3 ratio versus concentration for the (a) $EG_6$ peptide, (b) $EG_{12}$ peptide and (c) $EG_{24}$ peptide.

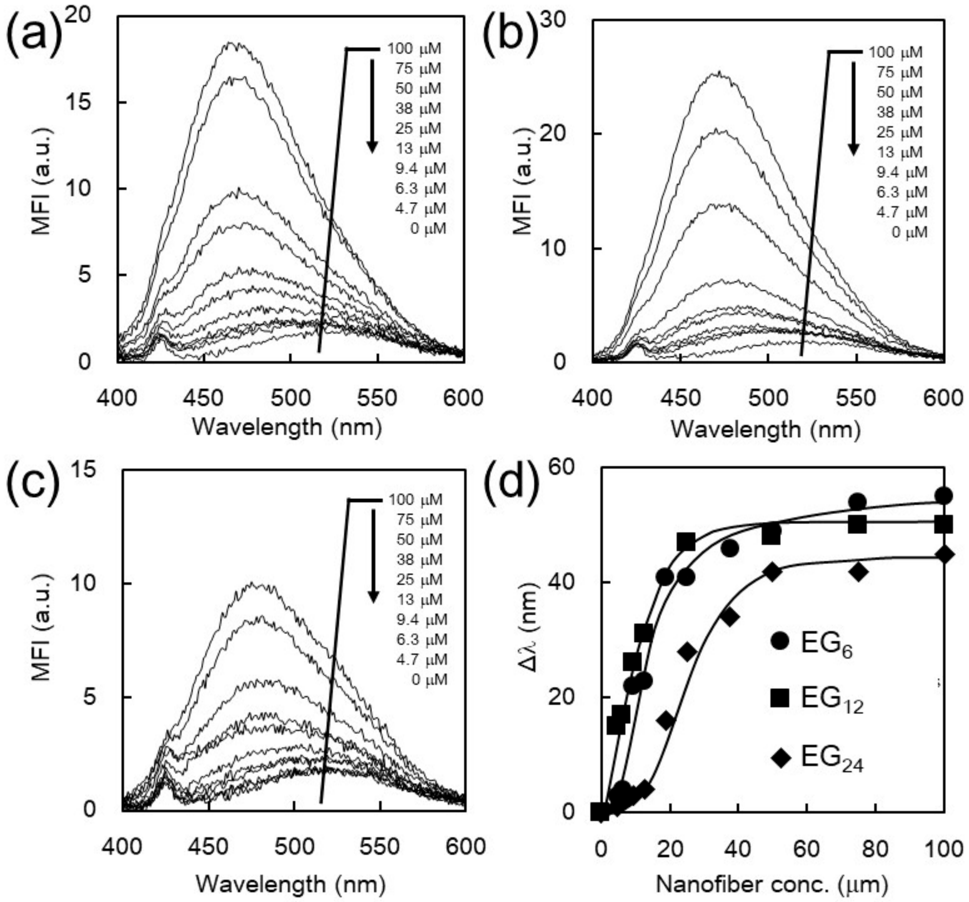


**Figure S3.** (a-c) Fluorescence spectra of ANS in the presence of (a) $EG_6$ NFs, (b) $EG_{12}$ NFs and (c) $EG_{24}$ NFs at concentrations of 10 μM in PBS. The concentration of the nanofibers ranged from 0 to 100 μM. (d) Shift in the wavelength peak maximum in fluorescence spectra of ANS (10 μM) in the presence of $EG_6$ NFs (circle), $EG_{12}$ NFs (square) and $EG_{24}$ NFs (diamond) at different peptide concentrations.

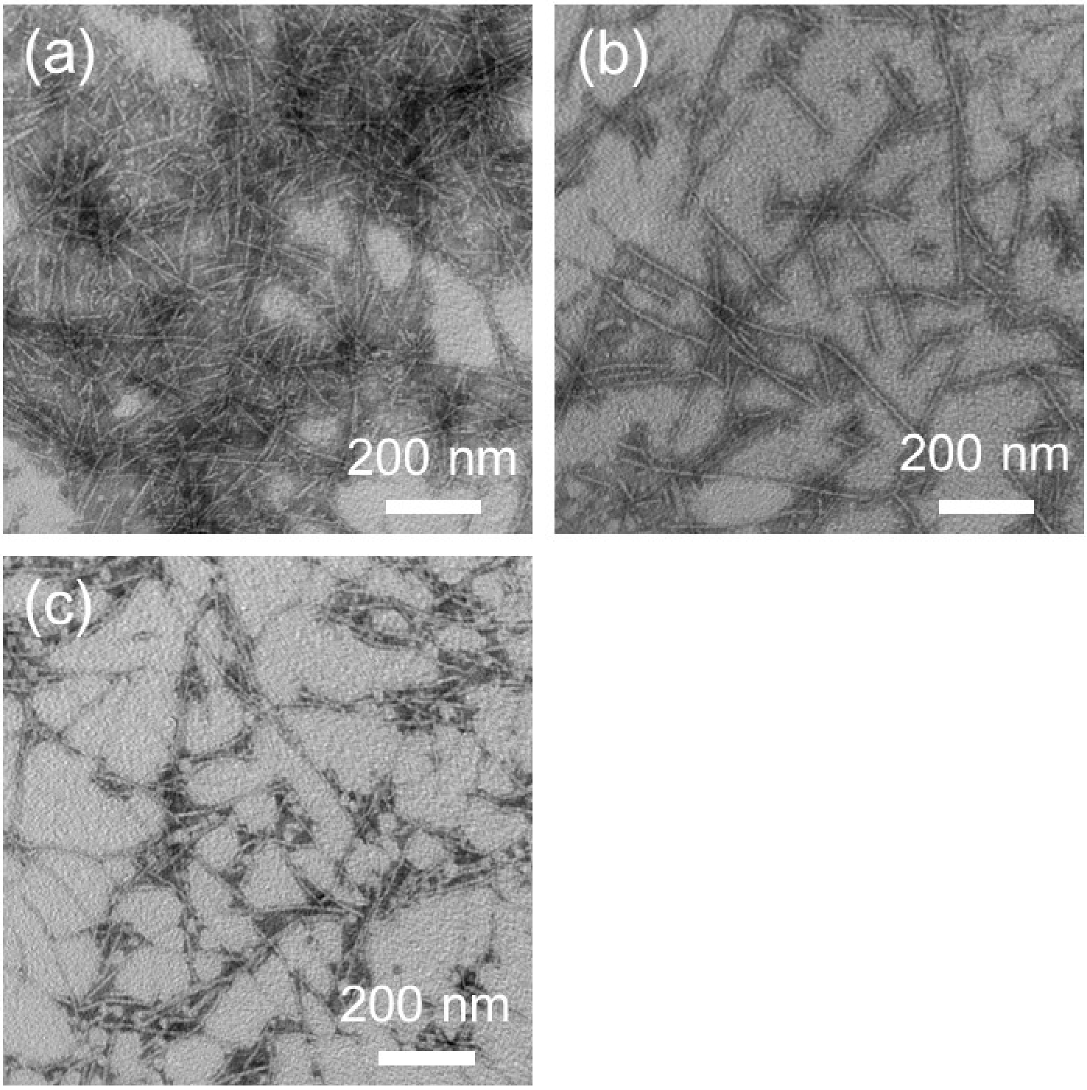


**Figure S4.** Negatively stained TEM images of short $EG_n$ NFs obtained by extrusion of nanofiber dispersions through a membrane filter with a pore diameter of 0.45 μm. (a) $EG_6$ NFs, (b) $EG_{12}$ NFs and (c) $EG_{24}$ NFs.

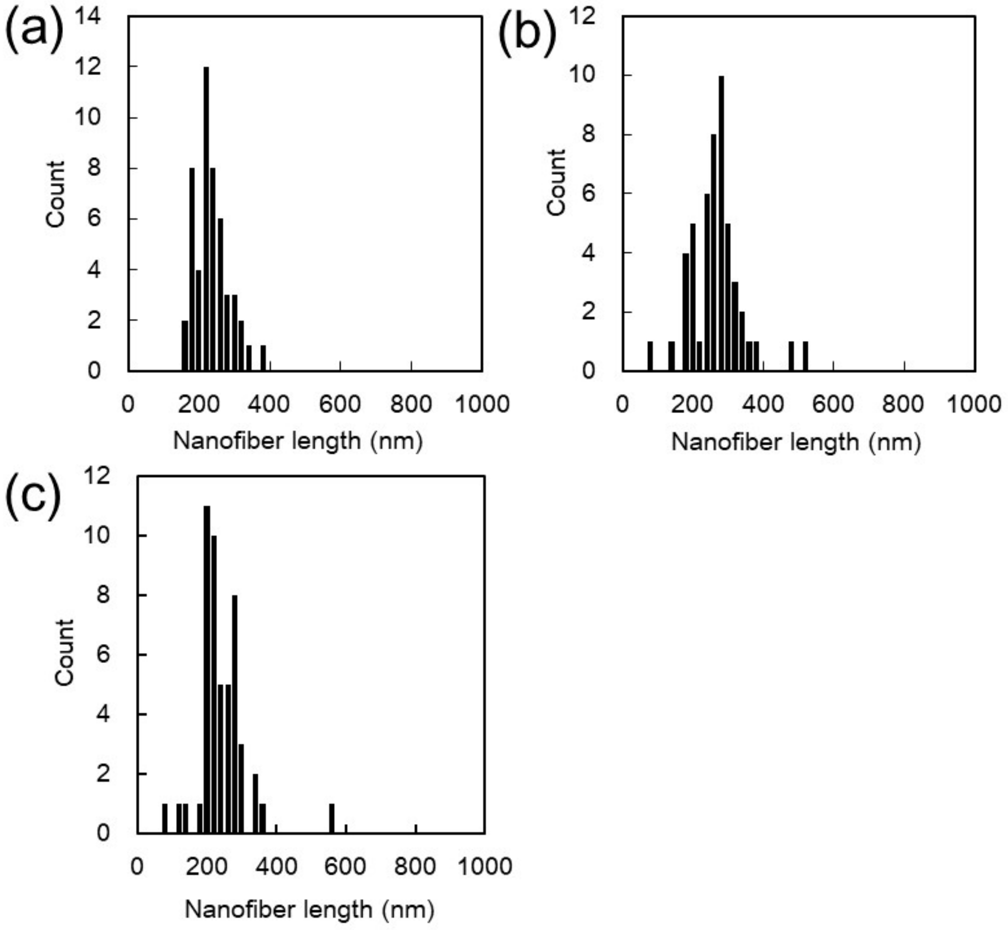


**Figure S5.** Size distribution of short $EG_n$ NFs estimated from TEM images shown in Figure S3. (a) $EG_6$ NFs, (b) $EG_{12}$ NFs and (c) $EG_{24}$ NFs.

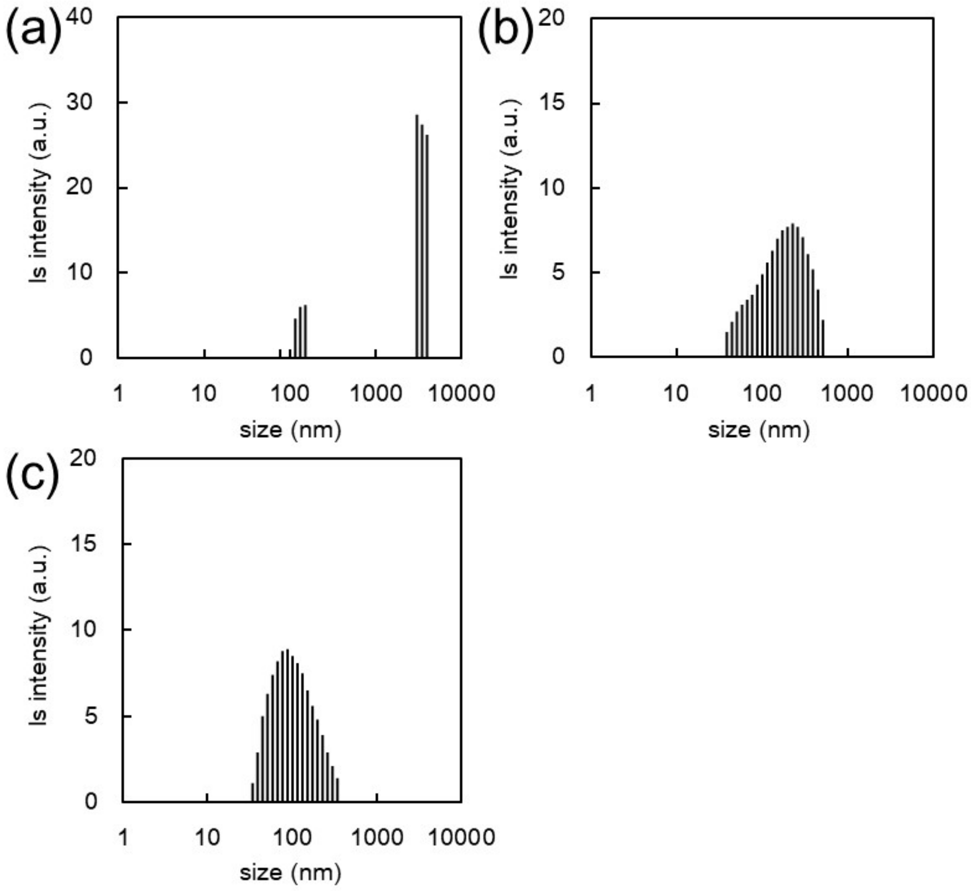


**Figure S6.** Size distribution of short $EG_n$ NFs in PBS measured by DLS. (a) $EG_6$ NFs, (b) $EG_{12}$ NFs and (c) $EG_{24}$ NFs.

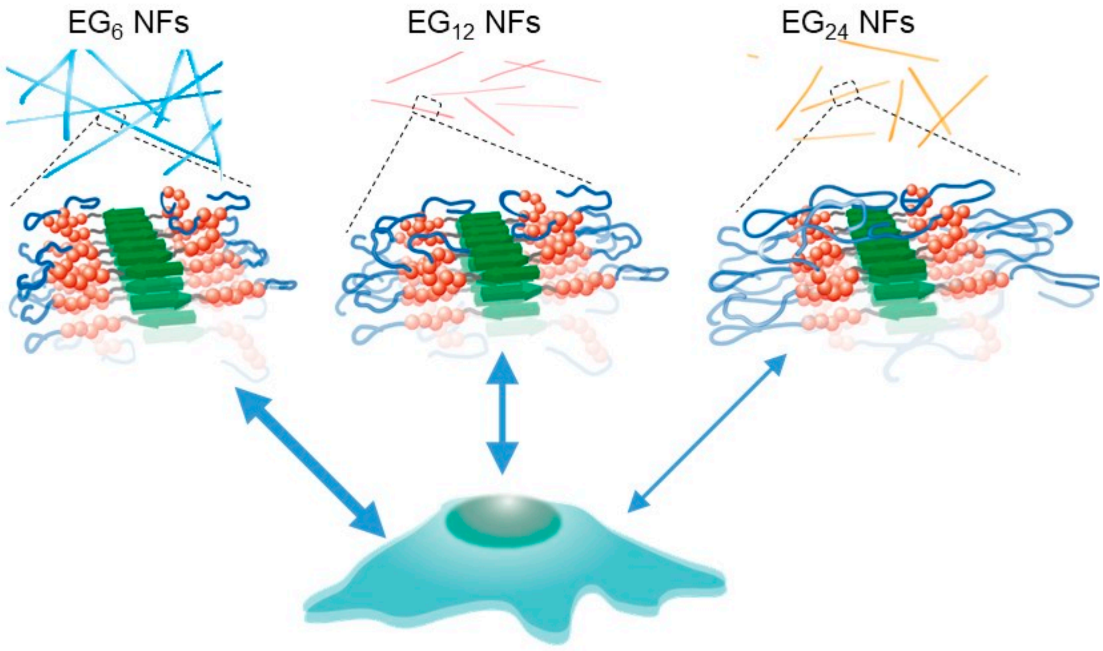


**Figure S7.** Schematic illustration of the interactions between $EG_n$ NFs and the cellular surface. Double head arrows represent the interactions and the width means the degree of them. Longer PEG chains inhibit interaction with cells, leading to low cellular uptake efficiency, low cytotoxicity and no immune-stimulation ability.
